# The involvement of Bcl-2 family proteins in AKT-regulated cell survival in cisplatin resistant epithelial ovarian cancer

**DOI:** 10.18632/oncotarget.13817

**Published:** 2016-12-07

**Authors:** Yan Dai, Shiguang Jin, Xueping Li, Daxin Wang

**Affiliations:** ^1^ The Second Xiangya Hospital of Central South University, Changsha, China; ^2^ Clinical Medical College, Yangzhou University, Yangzhou, China; ^3^ Medical Research Centre, Northern Jiangsu People's Hospital, Yangzhou, China; ^4^ Nanjing Hospital Affiliated to Nanjing Medical University, The First Hospital of Nanjing, Nanjing, China

**Keywords:** cisplatin, Bcl-2, AKT, epithelial ovarian cancer, drug resistance

## Abstract

Many studies involving patients with cisplatin-resistant ovarian cancer have shown that AKT activation leads to inhibition of apoptosis. The aim of this study was to examine the potential involvement of the Bcl-2 family proteins in AKT-regulated cell survival in response to cisplatin treatment. Cisplatin-sensitive (PEO1) and cisplatin-resistant (PEO4) cells were taken from ascites of patients with ovarian cancer before cisplatin treatment and after development of chemoresistance. It was found that cisplatin treatment activated the AKT signaling pathway and promoted cell proliferation in cisplatin-resistant EOC cells. When AKT was transfected into nucleus of cisplatin-resistant ovarian cancer cells, DNA-PK was phosphorylated at S473. The activated AKT (pAKT-S473) in these cells inhibited the death signal induced by cisplatin thereby inhibiting cisplatin-mediated apoptosis. Results from this study showed that the combination of cisplatin, DNA-PK inhibitor NU7441, and AKT inhibitor TCN can overcome drug resistance, increase apoptosis, and re-sensitize PEO4 cells to cisplatin treatment. A decrease in apoptotic activity was seen in PEO4 cells when Bad was downregulated by siRNA, which indicated that Bad promotes apoptosis in PEO4 cells. Use of the Bcl-2 inhibitor ABT-737 showed that ABT-737 binds to Bcl-2 but not Mcl-1 and releases Bax/Bak which leads to cell apoptosis. The combination of ABT-737 and cisplatin leads to a significant increase in the death of PEO1 and PEO4 cells. All together, these results indicate that Bcl-2 family proteins are regulators of drug resistance. The combination of cisplatin and Bcl-2 family protein inhibitor could be a strategy for the treatment of cisplatin-resistant ovarian cancer.

## INTRODUCTION

Ovarian cancer is a devastating malignancy, causing more deaths in the female US population compared to any other gynecologic cancer [1]. Of the three subgroups – epithelial, stromal, and germ cell tumors – majority of ovarian cancer cases are classified as epithelial carcinomas [1, 2]. Epithelial ovarian cancer (EOC) at first detection will already be at advanced stage in approximately 70% of diagnoses and of these, 30% of women will survive 5 years [1]. 80% of EOC patients will soon relapse after first-line chemotherapy [3]. This is in part due to the difficulty in diagnosis and treatment, particularly the rising concern of drug resistance. The median progression-free survival (PFS) is 18 months before most of these patients relapse [2]. Those with tumors that progress during or recur within 6 months of treatment are considered platinum-resistant [3]. Overall response rates with other treatments in these platinum-resistant patients are only 10–25% with relatively short durations of response [2]. Treatment failure is thought to be attributed to drug resistance in over 90% of cases with metastatic malignancy [2]. It can arise from multiple factors such as pharmacokinetic interactions, tumor micro-environment, and most likely, cancer-cell-specific abnormalities [2]. Therefore, there is a dire need to better elucidate the mechanisms behind ovarian cancer drug resistance and how to overcome them.

While prognosis is initially determined through the extent of initial surgical resection, chemotherapy is the cornerstone of maintenance of progression-free survival. Current standard drug treatment includes a combination regimen of paclitaxel and a platinum compound such as cisplatin [2]. The mechanism of action of cisplatin is DNA double-strand damage, which induces phosphatidylinositol 3-kinase (PI3K)/AKT activation downstream of DNA-PK [2, 4]. Hyper-activation of AKT is often seen in cisplatin-resistant epithelial ovarian cancers through the inhibition of p53 phosphorylation [5, 6]. Lee et al. demonstrated that the addition of an AKT inhibitor enhanced platinum-induced apoptosis in EOC cell lines [7]. Cisplatin-mediated cytotoxicity creates a downstream effect on a variety of molecular factors including activation of p53 and subsequent modulation of Bcl-2 family proteins including the pro-apoptotic proteins such as BAX and BAK, and anti-apoptotic proteins such as Bcl-2 and Mcl-1 [2, 8, 9]. Interestingly, AKT inhibition resulted in downregulation of Bcl-2 and upregulation of Mcl-1, suggesting a compensatory mechanism. Thus a more selective therapy was investigated by van Delft et al. who utilized a known Bcl-2 inhibitor, ABT-737 in mouse lymphoma cells [10]. Conversely, Bcl-2 can block p53-mediated apoptosis and is also a potential predictor of cisplatin-resistance in EOC [11]. Kassim et al. showed that overexpression of Bcl-2 correlated to decreased overall survival in ovarian cancer patients, therefore concluding the prognostic value of Bcl-2 in aggressive EOC [11]. Therefore, there may be abundant potential for Bcl-2 as a marker to aid in diagnosis and as an agent of overcoming cisplatin-resistance.

The aim of this study was to further examine the role of Bcl-2 family in AKT-regulated cell survival by evaluating how various known inhibitor compounds such as NU7441 (a DNA-PK inhibitor), TCN (an AKT inhibitor), and ABT-737 (a Bcl-2 inhibitor) affect cell apoptosis in cisplatin-sensitive and cisplatin-resistant EOC cells. Modulating this signaling pathway may help reverse drug resistance and reduce toxicity in these platinum-resistant patients, leading to novel EOC treatment methods.

## RESULTS

### Caspases activity remain constant under various drug treatments

**Figure 1:**
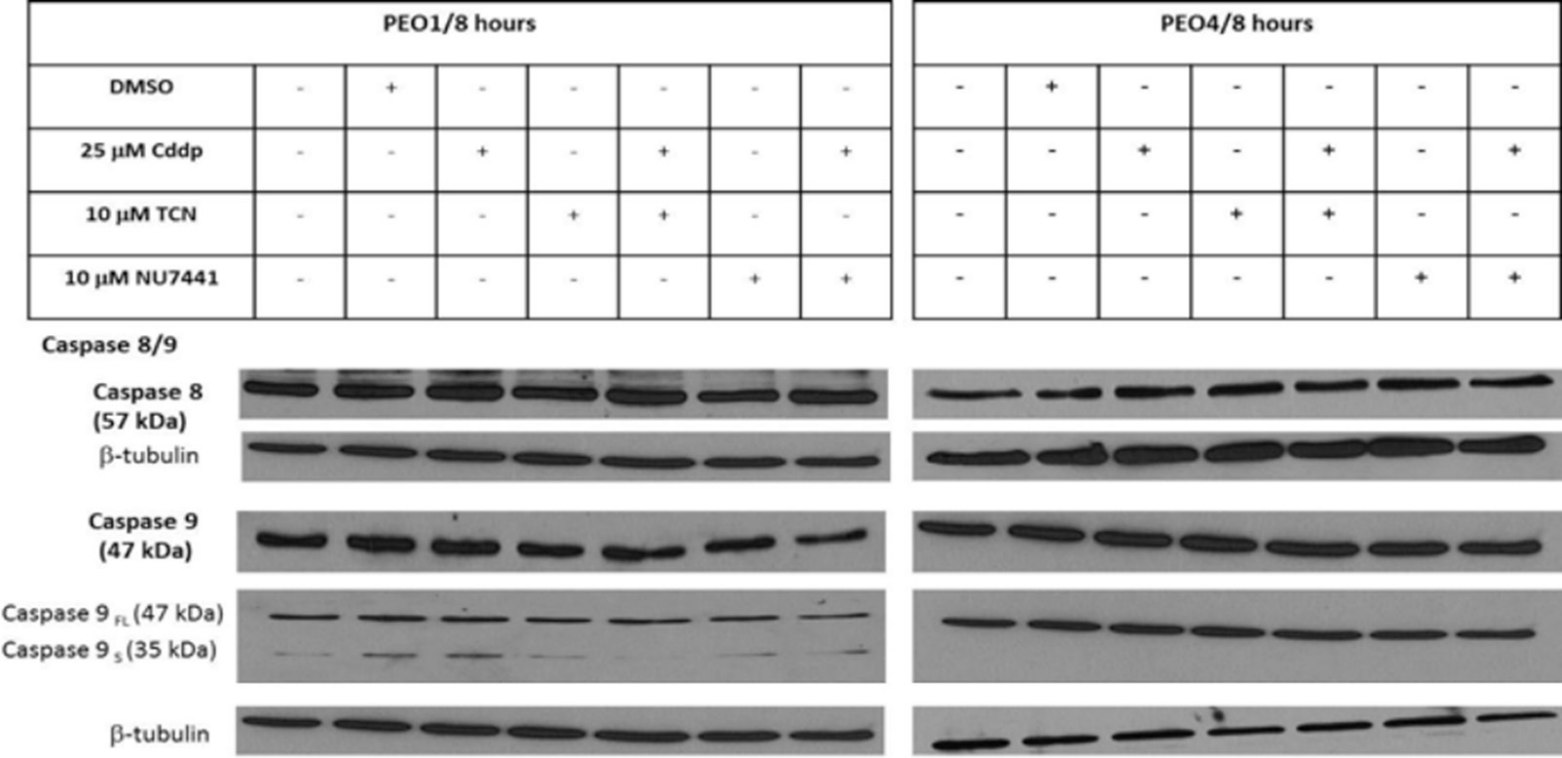
Expressions of caspase 8 and caspase 9 proteins in PEO1 and PEO4 cells in response to treatment with cisplatin, TCN and NU7441 Cisplatin-sensitive PEO1 cells and cisplatin-resistant PEO4 cells were treated as indicated. Cell lysates were prepared and were resolved by 12% SDS-PAGE agarose gel as described in Materials and Methods. The blots were probed with anti-caspase 8/9 antibodies and all blots were re-probed for b-tubulin expression as a loading control.

Caspase 8 and caspase 9 cleavage activation are crucial for the extrinsic and intrinsic apoptosis pathways, respectively [12]. Both caspase 8 and caspase 9 are important members of the cysteine aspartic acid protease family. Upon stimulation, the pro-caspase 9 (47 kDa) is associated with cytochrome c, forming apoptosome complex to process pro-caspase 9 into an active fragment (35 kDa or 17 kDa) [12]. Only the cleaved form of caspase 9 can further process other caspase members, including caspase 3 and caspase 7, to initiate caspase cascade and cell apoptosis. Caspase 8 was also activated by cleavage to induce apoptosis. In both PEO1 and PEO4 cells, caspase 8 did not change significantly in response to various drug treatments, including 10 μM TCN, 10 μM NU7441 and 25 μM cisplatin (Figure 1). Similarly, expression of full length of caspase 9 was not affected by drug treatment. However, the active cleaved form of caspase 9 (caspase 9s) was detected in PEO1 cells but not in PEO4 cells, and this was elevated in the presence of cisplatin. Although these results showed no clear role for caspase-8 or -9 in apoptosis in the response of PEO1 and PEO4 cells to cisplatin, they are downstream mediators of apoptosis and may be activated following treatment for a longer period (e.g. 24 hrs). Therefore, the expressions of the Bcl-2 family proteins, which are upstream of these caspases were next examined (Table 1).

**Table 1:** Validated antibodies used for Western blotting (WB) experiments

Antibody	Dilutions	Solvent	Supplier	Molecular Weight
pAKT (Ser473) (Rabbit)	1:1000	5% BSA in TBST	Cell Signaling	60 kDa
pSrc (Tyr416) (Rabbit)	1:1000	5% BSA in TBST	Cell Signaling	60 kDa
Cyclin B1 (Mouse mAb)	1:250	5% milk in TBST	Santa Cruz	60 kDa
**Pro-apoptotic Bcl-2 family proteins and Caspases**				
Bax (Rabbit mAb)	1:1000	5% milk in TBST	Cell Signaling	20 kDa
Bak (Rabbit mAb)	1:1000	5% milk in TBST	Cell Signaling	25 kDa
Bim (Rabbit mAb)	1:1000	5% milk in TBST	Cell Signaling	12, 15, 23 kDa
BID (Human specific)	1:1000	5% milk in TBST	Cell Signaling	15,22 kDa
Puma (Rabbit Ab)	1:1000	5% milk in TBST	Cell Signaling	23 kDa
Bik (Rabbit Ab)	1:1000	5% milk in TBST	Cell Signaling	20 kDa
Bad (Rabbit mAb)	1:1000	5% milk in TBST	Cell Signaling	23 kDa
P-Bad (Ser 136) (Rabbit)	1:1000	5% BSA in TBST	Cell Signaling	23 kDa
Caspase 8 (Mouse mAb)	1:1000	5% milk in TBST	Cell Signaling	18, 43, 57 kDa
Caspase 9 (Rabbit)	1:1000	5% milk in TBST	Cell Signaling	17, 35, 37, 47 kDa
**Anti-apoptotic Bcl-2 family proteins**				
pBcl-2 (Ser70) (Rabbit)	1:1000	5% BSA in TBST	Cell Signaling	28 kDa
pBcl-2 (Thr56) (Rabbit)	1:1000	5% BSA in TBST	Cell Signaling	28 kDa
Bcl-2	1:1000	5% milk in TBST	Cell Signaling	26 kDa
XIAP	1:1000	5% milk in TBST	Cell Signaling	53 kDa
Mcl-1 (Rabbit mAb)	1:1000	5% milk in TBST	Cell Signaling	40,35 kDa
Bcl-XL (Rabbit mAb)	1:1000	5% milk in TBST	Cell Signaling	30 kDa
				
β-tubulin	1:5000	5% milk in TBST	Santa Cruz	55 kDa
Anti-rabbit-HRP	1:2000	5% milk in TBST	Dako	NA
Anti-mouse-HRP	1:5000	5% milk in TBST	Dako	NA

All antibodies are diluted as specified in the table. p-, phosphor-; mAb, monoclonal antibody; pAb, polyclonal antibody; NA, not applicable.

### Expression of pro-apoptotic Bcl-2 family proteins are altered differentially in cisplatin-sensitive and –resistant EOC cells in response to treatment with AKT and DNA-PK inhibitors

Bcl-2 family proteins are essential molecules involved in the intrinsic apoptosis pathway and may be downstream targets for AKT activation, therefore contributing to the development of cisplatin-resistance. Identification of such targets could provide clues for drug discovery in reversing the cisplatin-resistance in EOC.

**Figure 2:**
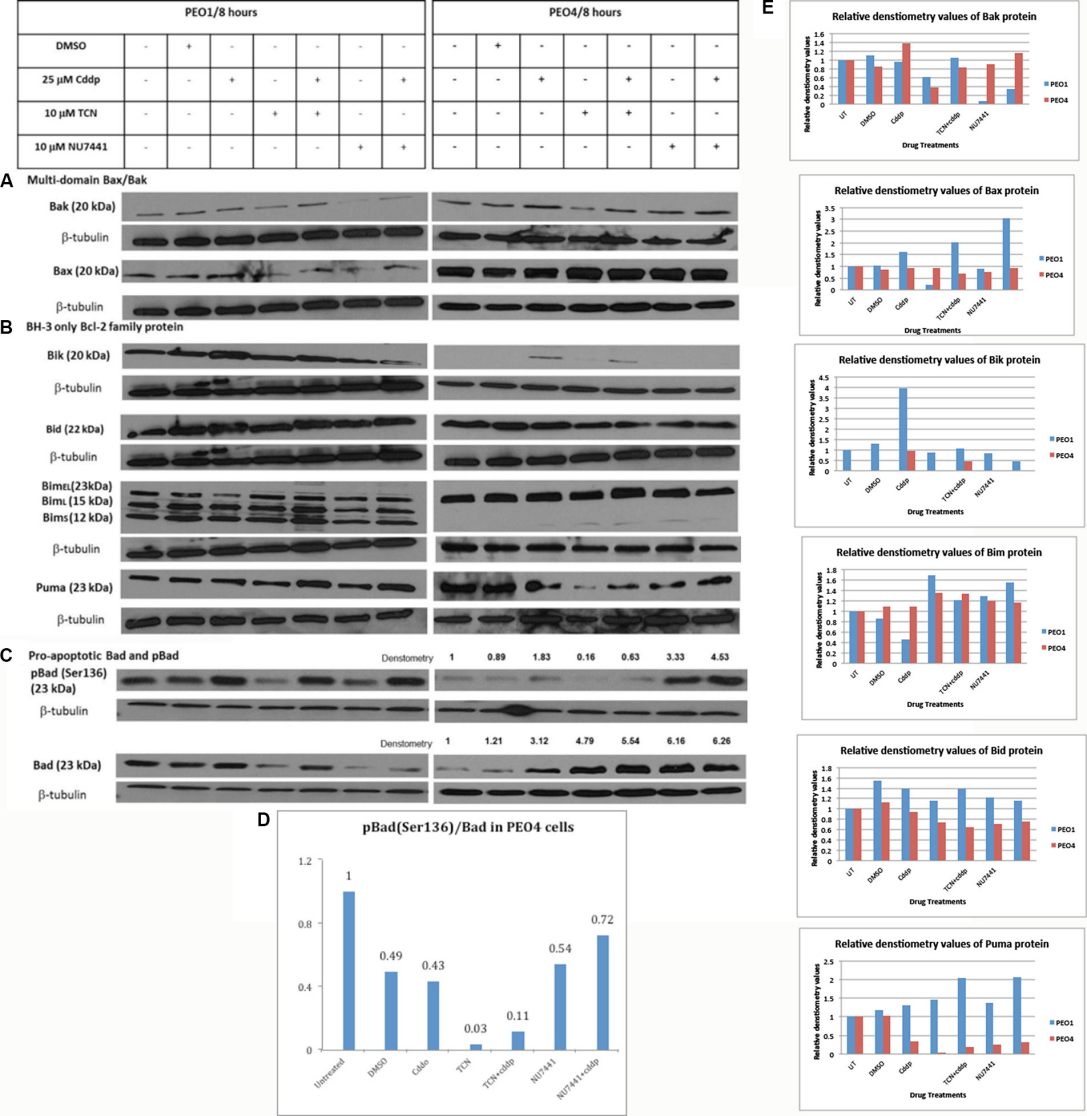
Expression of pro-apoptotic Bcl-2 family proteins in PEO1 and PEO4 cells in response to treatment with cisplatin, TCN and NU7441 Cisplatin-sensitive PEO1 and cisplatin—resistant PEO4 cells were treated as indicated. Cell lysates were prepared and were separated by 12% SDS-PAGE gel as described in Materials and Methods. Western Blotting was carried out to examine expression of (**A**) multi-domain pro-apoptotic Bax and Bak (**B**) pro-apoptotic BH-3 only Bcl-2 family proteins (**C**) total and phosphorylated pro-apoptotic Bad. (**D** and **E**) Densitometry values for protein expression in PEO4 cells analyzed by Image J software and normalized to their corresponding b-tubulin densities.

Following the activation of intrinsic apoptotic pathway, formation of Bax and Bak oligomers at the outer mitochondrial membrane increases the permeability of this membrane, permitting the release of pro-apoptotic mediators such as cytochrome c [12]. Therefore Bax and Bak are crucial for this pathway and whether AKT inhibition in combination with cisplatin therapy stimulates the Bax/Bak oligomerization directly or indirectly is still unknown. To investigate the potential role of a Bax/Bak-mediated mechanism underlying reversal of cisplatin resistance in response to AKT inhibition, the expression levels of Bax and Bak were examined using Western blotting (Table 1) (Figure 2A) and the densities of the proteins were measured and normalized to their corresponding beta-tubulin densities (Table 2).

**Table 2:** The densities of protein bands were measured and normalized to corresponding β-tubulin expression to compare the alterations in expressions in response to AKT inhibition and cisplatin therapy and their combination

	PEO1							PEO4						
	UT	DMSO	Cddp	TCN	TCN+cddp	NU7441	NU7441+cddp	UT	DMSO	Cddp	TCN	TCN+cddp	NU7441	NU7441+cddp
**Bak**	++	++	++	+	++	+	−	+	+	++	−	+	+	+
**Bax**	+	+	++	−	++	−	++	+	+	+	++	++	++	++
**Bik**	+	+	+++	+	++	+	−	−	−	++	−	+	−	−
**Bid**	+	++	++	+	++	+	++	++	++	++	+	+	+	+
**BimEL**	+	+	−	+	+	+	+	+	+	+	+	++	+	+
**Puma**	+	+	+	+	++	+	++	++	++	+	−	−	−	+
**pBad(Ser136)**	+	+	++	−	+	−	+	+	+	++	−	−	+++	++++
**Bad**	++	++	+++	+	++	−	+	−	−	++	+++	++++	+++++	+++++
**pBcl-2 (Ser70)**	++	++	+	−	−	−	−	−	−	−	−	−	−	−
**Bcl-2**	+	+	+	++	+	−	−	++	++	+	+	−	−	−
**Mcl-1**	++	++	++	++	++	+	++	+	+	−	−	++	++	++
**Bcl-XL**	++	++	++	++	++	+	+	++	++	++	−	−	+	+
**XIAP**	++	++	−	−	+	−	+	+	+	+	−	+	−	+

In this table “+” stands for the increase in expression whereas “–“ indicates the inhibition of this protein expression. UT, untreated; cddp, 25 μM cisplatin; TCN, 10 μM TCN; NU7441, 10 μM NU7441.

In PEO1 cells Bak expression was found to be decreased when the cells were treated for 8 hrs with 10 μM AKT inhibitor TCN, and this decrease was prevented when the cells were co-treated with 25 μM cisplatin. Bak expression was also reduced, although to a greater extent, when cells were treated for 8 hrs with 10 μM DNA-PK inhibitor NU7441 alone. This was only partially reversed when cells were treated with 10 μM in combination with 25 μM cisplatin. In comparison, expression of Bak was only decreased in PEO4 cells following treatment with 10 μM TCN for 8 hrs. This alluded that TCN-mediated AKT inhibition could decrease the Bak expression and this suppression may be reversed by cisplatin-induced DNA damage. On the other hand, Bax expression was also decreased by 10 μM NU7441 and 10 μM TCN for 8 hrs, although the presence of cisplatin suppressed the inhibitory effect exerted by these two inhibitors. In PEO4 cells, TCN and NU7441, either alone or in combination with cisplatin enhanced Bax expression. The upregulation of Bax may be associated with AKT inhibition and DNA-PK inhibition, implying that Bax rather than Bak could become a potential therapeutic strategy.

Inhibition of pro-apoptotic BH-3 only proteins is also possible to be involved in AKT-promoted cell survival signaling because of their close association with other Bcl-2 family members. Thus the expression of pro-apoptotic BH-3 only proteins in PEO1 and PEO4 cells was also evaluated. Expression of Bcl-2 interacting killer (Bik) protein in cisplatin-sensitive PEO1 cell line was found to be increased following treatment with 25 μM cisplatin (Figure 2B). This increase occurred to a lesser extent when the cells were co-treated with TCN, and was prevented in cells co-treated with NU7441. In comparison, basal expression of Bik was not detected in cisplatin-resistant PEO4 cells. However, 25 μM cisplatin treatment in PEO4 cells induced a low Bik expression and TCN or NU7441 treatments prevented this induction completely.

Another pro-apoptotic BH-3 only protein Bid, exhibited completely different expression patterns in PEO1 and PEO4 cells. Bid expression was not changed in PEO1 cells under all conditions but a slight decrease was seen in PEO4 cells treated with TCN, TCN/cisplatin, NU7441 and NU7441/cisplatin (Figure 2B). Moreover, Bid expression was not stimulated by AKT inhibition directly. From these evidences we assumed that Bid could be a natural killer in cisplatin-sensitive PEO1 cells but not in cisplatin-resistant PEO4 cells due to its high endogenous expression in PEO1 cells only.

Bim has shown distinct expression patterns in PEO1 and PEO4 cells. There are three isoforms of the BH3-only protein Bim: BimEL (23 kDa), BimL (15 kDa) and BimS (12 kDa). BimS is the most cytotoxic isoform and is only transiently expressed during apoptosis [13]. In contrast, the apoptotic activity of the longer isoforms may be inhibited by phosphorylation and normally the BimEL and BimL are sequestered dynein motor complex and only released during apoptosis [13]. In PEO1 cells, three bands were observed: the top band representing the full length of Bim (BimEL 23 kDa) while the other two bands indicated the two additional isoforms of Bim (BimL: 15 kDa and BimS: 12 kDa) (Figure 2B). In PEO4 cells, expression of BimEL was found to be increased after co-treatment with 10 μM TCN and 25 μM cisplatin. In addition, a slight increase in expression of BimL was detected in cells treated with 10 μM TCN and 10 μM NU7441, both alone and in combination with cisplatin. More importantly, the changes of the short forms of Bim between PEO1 and PEO4 cells were striking. The evidence presented here may suggest that Bim in PEO1 is poised for apoptosis whereas that in PEO4 is not to the same extent.

Puma, a pro-apoptotic BH-3 only Bcl-2 family protein identified recently, was also screened in this study [15]. Puma was increased in PEO1 cells treated with 10 μM TCN or 10 μM NU7441 in combination with 25 μM cisplatin at the same time (Figure 2B). In contrast, TCN and NU7441 treatments repressed Puma expression in PEO4 cells in the absence of cisplatin but this inhibitory effect was abrogated when these compounds were combined with cisplatin treatment. AKT inhibition, as a result, may interfere with puma expression in cisplatin-sensitive PEO1 cells but not in its cisplatin-resistant counterpart PEO4 cells.

### Bad is involved in the responses of EOC cells to cisplatin

The active form of AKT phosphorylates Bad at Ser136 residue, directly inhibiting cell apoptosis and decreasing the chemo-sensitivity to cisplatin in EOC cells [16]. Therefore Bad protein expression and pBad (Ser136) expression levels before and after AKT inhibition in the presence or absence of cisplatin were of particular interest and were investigated to determine the correlation between Bad activation (i.e. phosphorylation) and AKT inhibition. In cisplatin-sensitive PEO1 cells, treatment with 10 μM TCN exerted a significant inhibitory effect on Bad expression and co-treatment with 25 μM cisplatin restored the expression to the basal level, which was observed in the untreated PEO1 cells (Figure 2C). Moreover, treatment with 10 μM NU7441 mediated a slightly greater inhibitory effect on Bad expression, compared with TCN alone, but the combination of cisplatin and NU7441 did not restore Bad expression. On the other hand, pBad (Ser136) expression was greatly increased in PEO1 cells in response to treatment with 25 μM cisplatin. This was not prevented in cells co-treated with 10 μM TCN or 10 μM NU7441, although treatment with these agents alone decreased expression of pBad (Ser136) in these cells. This indicated that Bad inactivation by AKT was not influenced by AKT signaling directly. In other words, the AKT inhibition resulted from direct inhibition by TCN or indirect inhibition by NU7441 did not interfere with the Bad inactivation in cisplatin-sensitive PEO1 cells (Figure 2C).

In contrast, Bad expression was increased 3-fold in PEO4 cells treated with 25 μM cisplatin (Figure 2C). Moreover, 10 μM TCN and 10 μM NU7441 treatments also enhanced Bad expression by 4.8- and 6-fold, respectively. Cisplatin used in the presence of TCN enhanced the Bad expression further, with a 5.5-fold increase compare with basal levels measured. In comparison, Bad protein in PEO4 cells treated with the combination of NU7441 and cisplatin presented a similar increase in expression compared with those treated with NU7441 alone (fold-increases of 6.16 and 6.26, respectively) (Figure 2C). Expression of pBad (Ser136) was slightly increased in PEO4 cells treated with 25 μM cisplatin. However, a 3-fold increase in expression was detected in these cells following treatment with 10 μM NU7441, and this up-regulation increased to 4.5-fold when NU7441 was combined with cisplatin. pBad (Ser136)/Bad ratios in PEO4 cells were obtained by dividing the densitometry values of pBad (S136) to the corresponding densitometry values of total Bad (Figure 2D). It is obvious that pBad was rarely presented in PEO4 cells and cisplatin did not affect the phosphorylation of Bad due to the similar values of pBad/Bad was obtained in presence of cisplatin (0.43) in comparison to that in cells incubated with DMSO control (0.49). In comparison, pBad/Bad ratios obtained from PEO4 cells when they were treated with individual TCN or TCN in combination with cisplatin were relatively low (0.03 and 0.11, respectively), indicating that AKT mediated the inhibitory Bad phosphorylation at Ser136 (Figure 2D). Nevertheless, individual use of NU7441 or its combination with cisplatin in PEO4 cells increased pBad levels significantly and the pBad/Bad values were 0.54 and 0.72 under these two conditions. This implies that the DNA-PK inhibition does not fully mimic AKT inhibition in terms of the modulation of pro-apoptotic proteins (Figure 2E).

The increased expression of total Bad in response to TCN and NU7441 clearly suggests that this could be an important mechanism by which AKT inhibition re-sensitizes cisplatin-resistant PEO4 cells to cisplatin. To investigate this further, Bad siRNA knockdown transfection in PEO4 cells was carried out in the rest of study.

### Anti-apoptotic Bcl-2 protein is an important modulator of chemosensitivity in EOC cells

**Figure 3:**
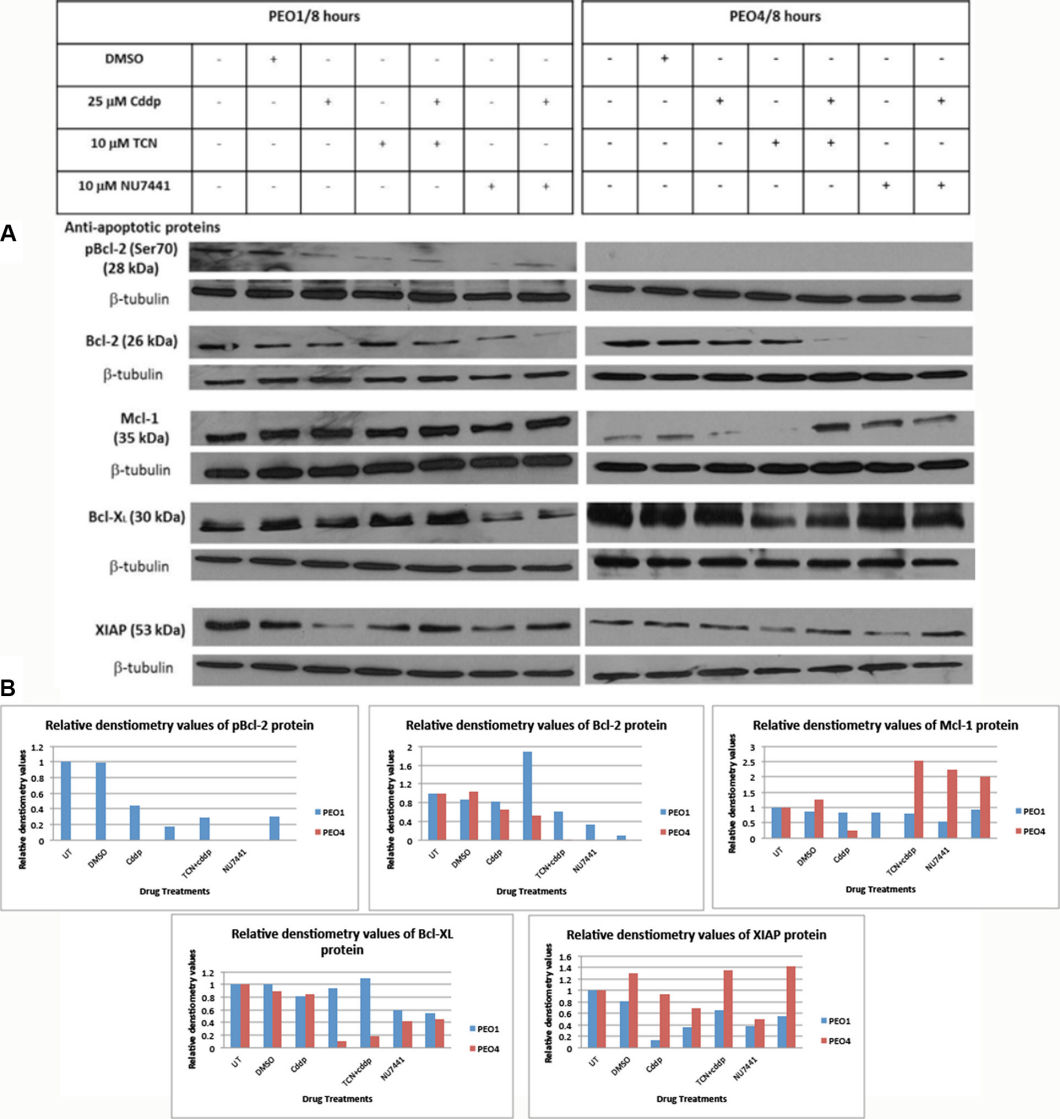
Expression of anti-apoptotic Bcl-2 family proteins in PEO1 and PEO4 cells in response to treatment with cisplatin, TCN and NU7441 Cisplatin-sensitive PEO1 and cisplatin-resistant PEO4 cells were treated as indicated. Cell lysates were prepared and were separated by 12% SDS-PAGE gel as described in Materials and Methods. (**A**) Blots were probed with anti-apoptotic pBcl-2 (Ser70), total Bcl-2, Mcl-1, Bcl-XL, and XIAP and all membranes were incubated with β-tubulin, which was used as a loading control. (**B**) Densitometry values for protein expression in PEO4 cells analyzed by Image J software and normalized to their corresponding β-tubulin densities.

Bcl-2 was the first identified anti-apoptotic protein and is known to inhibit apoptosis by sequestering its pro-apoptotic partners. Bcl-2 is frequently phosphorylated via several sites, including Thr56, Ser70, Thr74 and Ser87. Ser70 phosphorylation is the most frequently observed, suggesting its functional significance and phosphorylation of Bcl-2 is assumed to abrogate the pro-survival activity of Bcl-2 (reviewed in [17]). Therefore the expression of total Bcl-2 and pBcl-2 were tested here to examine the potential involvement of Bcl-2 in AKT-promoted cell survival signaling. As shown in Figure 3, total Bcl-2 expression exhibited a relative low basal level in PEO1 cells and cisplatin did not affect Bcl-2 levels. The changes of Bcl-2 expression were relatively modest except for a decrease in cells treated with combination of NU7441 and cisplatin (Figure 3).

On the other hand, in cisplatin-resistant PEO4 cells, Bcl-2 protein expression was not affected by cisplatin treatment but was completely abrogated by NU7441 treatments. Moreover, this decrease in Bcl-2 protein expression was maintained in cells treated with NU7441 and cisplatin at the same time. TCN inhibitor alone did not affect Bcl-2 protein expression, whereas TCN used in the presence of cisplatin suppressed Bcl-2 protein expression extensively (Figure 3). This provides evidence that AKT inhibition by NU7441 and TCN had a significant inhibitory impact on anti-apoptotic Bcl-2 expression, especially in combination with cisplatin therapy. Thus TCN/NU7441 could become good suppressive agents targeting Bcl-2 protein.

The phosphorylation status of Bcl-2 was also investigated using anti-pBcl2 (Ser70) and anti-pBcl (Thr56) antibodies. pBcl-2 (Thr56) was not detected in either PEO1 or PEO4 cells whereas pBcl-2 (Ser70) was found in PEO1 cells only but not in PEO4 cells. pBcl-2 (Ser70) was only observed in control cells (no treatment and DMSO treated) and was reduced in all other treatments and in particular by NU7441 treatment where it was barely detectable. Cisplatin appeared to have some modest restorative effect on Bcl-2 levels in combination with either TCN or NU7441 (Figure 3).

Mcl-1 is another anti-apoptotic protein within the Bcl-2 family proteins and localizes to the mitochondria, interacting with and antagonizing pro-apoptotic Bcl-2 family members [18]. Mcl-1 can be activated at both transcriptional and post-transcriptional level, which is distinct from other family members that tend to be activated at protein level [18]. Mcl-1 is rapidly transcribed via the PI3K/AKT signaling pathway, leading to the increased expression during myeloid differentiation and cytokine stimulation [18]. Mcl-1 expression level in PEO1 cells did not exhibit significant differences among various drug treatments (Figure 3). In PEO4 cells, Mcl-1 expression was decreased following treatment with cisplatin and TCN. However, when a combination of TCN with cisplatin was used, Mcl-1 expression increased greatly (Figure 3). NU7441 alone increased Mcl-1 expression in PEO4 cells, although combination with cisplatin modestly reduced this effect. Considering the expression variances of Bcl-2 and Mcl-1 in PEO4 cells, it is possible that the increased Mcl-1 expression was induced by the loss of Bcl-2 expression in response to AKT inhibition, utilizing as a potential compensatory mechanism. Therefore, a specific inhibitor for Bcl-2, Bcl-XL and Bcl-w, termed BH-3 mimetic ABT-737 inhibitor, could be used in PEO1 and PEO4 cells to evaluate the correlation between Bcl-2 and Mcl-1 in determining the chemosensitivity.

Bcl-XL is also a member of anti-apoptotic Bcl-2 family proteins and it prevents apoptosis via two distinct mechanisms, heterodimerization with pro-apoptotic proteins and formation of mitochondrial outer membrane pores to help maintain a normal membrane state under stressful conditions [19]. Bcl-XL expression in PEO1 cells did not vary under different drug treatments with the exception of NU7441 (Figure 3). NU7441 treatment reduced Bcl-XL expression both in the presence or absence of cisplatin. However, NU7441 did not have a similar impact on Bcl-XL expression in PEO4 cells and Bcl-XL expression was only suppressed to some extent by TCN and its combination with cisplatin (Figure 3). The divergent effects on Bcl-XL expression exerted by TCN- and NU7441-mediated AKT inhibition suggested that inhibition of Bcl-XL expression may not be involved in DNA-damage mediated AKT signaling pathway.

In addition to anti-apoptotic Bcl-2 family proteins, XIAP (X-linked inhibitor of apoptosis protein) may be a key determinant in chemosensitivity by suppressing the apoptotic activities induced by cisplatin in ovarian cancer [20]. XIAP is suggested as a activator in PI3K/AKT survival pathway in chemo-sensitive and chemo-resistant ovarian cancer cell lines [20]. Here we investigated the XIAP expression levels under specified conditions and there are negligible differences on XIAP expression across the various conditions. However, XIAP expression level was clearly reduced upon cisplatin treatment in PEO1 cells but not in PEO4 cells (Figure 3). XIAP expression was also reduced by TCN and NU7441 in both PEO1 and PEO4 cells, indicating a potential correlation between XIAP expression and AKT activation regardless of the extent of chemo-sensitivity of the cells. However, the repression of XIAP protein was restored by cisplatin treatments in PEO1 and PEO4 cells (Figure 3). In all, XIAP demonstrated similar expression patterns in PEO1 and PEO4 cells in response to individual use of TCN and NU7441 as well as their combinations with cisplatin.

### Bad siRNA knockdown partially suppressed the cisplatin-inducted apoptosis

**Figure 4:**
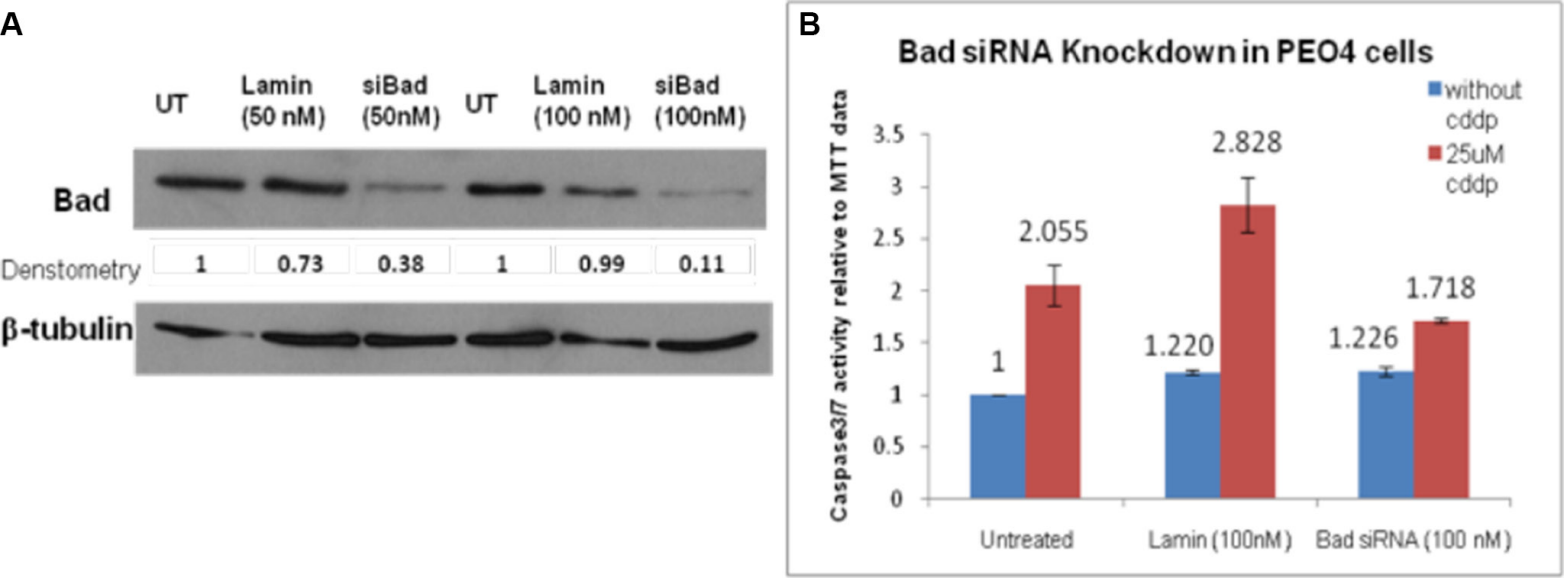
(**A**) siRNA knockdown of Bad in PEO4 cells. PEO4 cells were treated with Bad siRNA or Lamin control siRNA, and expression of total Bad were assessed by Western Blotting as described in Materials and Methods. 90% knockdown of Bad was achieved when PEO4 cells were transfected with Bad siRNA (100 nM). The apoptotic activity induced by cisplatin was significantly reduced by Bad siRNA when compared to that in untreated cells. (**B**) Bad siRNA knockdown repressed the apoptotic activity of cisplatin in chemo-resistant PEO4 cells. The caspase3/7 assay and MTT assay were performed to observe the influence of Bad knockdown on apoptosis in response to 25 μM cisplatin treatment. siGenome Lamin A/C control was used as an experimental control and the Lamin transfection did not affect the apoptotic response of PEO4 cells to cisplatin.

Bad had been demonstrated as a potential downstream target for AKT activation and the influence of Bad knockdown on cisplatin-induced apoptotic activity in PEO4 cells was examined by Bad siRNA transfection, caspase 3/7 and MTT assays. Firstly, the working concentration of Bad siRNA was optimized to achieve the best transfection efficiency with minimal cytotoxicity. Western blotting analysis was then carried out to assess efficiency of Bad siRNA knockdown, showing that 50 nM Bad siRNA knocked down Bad expression by 50% in comparison to 50 nM siGenome Lamin A/C control (Figure 4A). Furthermore, Bad siRNA at 100 nM inhibited Bad protein expression by 90% and thus Bad siRNA (100 nM) was adopted for the further experiments (Figure 4A).

To investigate the apoptotic activity after transfection, the Bad siRNA-transfected PEO4 cells were seeded in 96 well plates for caspase 3/7 and MTT assays. As is widely known, caspase3/7 assay provides a proluminescent caspase 3/7 substrate, releasing aminoluciferin by cleavage [21]. The cleaved aminoluciferin can be consumed by the luciferase, producing a luminescent signal which is proportional to the caspase 3/7 activity [21]. As shown in Figure 4B, the induction of apoptosis on cisplatin treatment (1.4-fold increase: 1.718/1.226) was reduced in Bad siRNA-transfected PEO4 cells when compared to that in untreated PEO4 cells (2.055-fold increase) and siGenome Lamin-transfected control cells (2.31-fold increase) (Figure 4B). Although this indicates that Bad is functionally pro-apoptotic in response to cisplatin in cisplatin-resistant PEO4 cells, the incomplete inhibition of apoptosis suggests that Bad was not solely responsible for the apoptotic responses in PEO4 cells.

### ABT-737 sensitized PEO1 and PEO4 cells to cisplatin treatment

**Figure 5:**
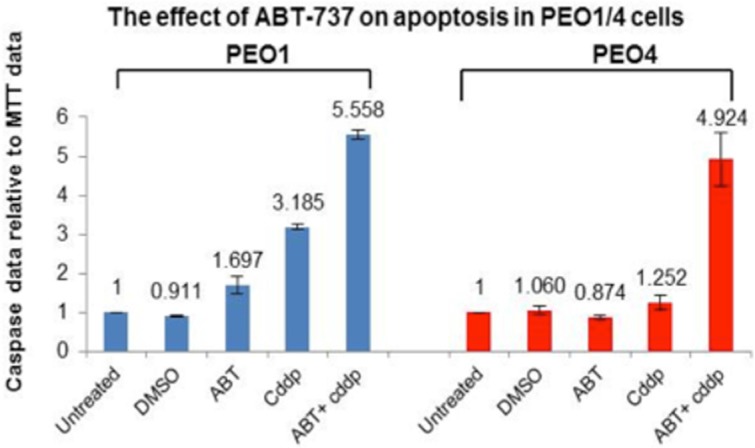
The effect of ABT-737 inhibitor on cisplatin-induced apoptosis in PEO1 and PEO4 cells A small molecule BH-3 mimetic inhibitor ABT-737 (1 μM), which specifically inhibits Bcl-2 and Bcl-XL, was used to treat PEO1 and PEO4 cells individually or in combination with 25 μM cddp. After 8-hr incubation, ABT-737 inhibitor increased the chemo-sensitivity of PEO1 and PEO4 cells to cddp to a high level compared to their corresponding basal levels (25 μM cddp).

BH-3 mimetic ABT-737 is a small molecule inhibitor that could trigger Bax/Bak-mediated apoptosis and it has a high affinity for Bcl-2, Bcl-XL and Bcl-w but not Mcl-1 [22]. Moreover, the inability of ABT-737 to target Mcl-1 and the enhancement of Mcl-1 expression conferred resistance in cancer cells [22]. Here we investigate the potential of ABT-737 in reversing the resistance by targeting the Bcl-2 and Bcl-XL in ovarian cancer cell lines via caspase3/7 and MTT assays. Here ABT-737 (1 mM) was found to sensitize the PEO1 and PEO cells to cisplatin to a large extent (1.85 fold increases in PEO1 cells and 3.93 fold increases in PEO4 cells) (Figure 5). Importantly, ABT-737 alone was relatively non-apoptotic towards both cell lines (1.697 and 0.874 in PEO1 and PEO4, respectively, implying that ABT-737 did not confer much toxicity to these two cell lines and without an apoptotic stimulus (e.g. cisplatin) the cells would not induce apoptosis even when anti-apoptotic proteins are inhibited (Figure 5). These results suggest that ABT-737 could be a promising therapeutic agent for reversing the cisplatin-resistance in ovarian cancer and can be used in combination with cisplatin in advanced EOC. Although Mcl-1 expression was increased in response to TCN/NU7441-based treatments in PEO4 cells, this elevation was not sufficient to overcome the inhibition of Bcl-2 protein under the same conditions (Figure 3). In this case, the combination therapy of ABT-737 and AKT inhibitors like TCN and NU7441 is of profound importance to be investigated furthermore due to the expression manners observed in PEO4 cells.

## DISCUSSION

Ovarian cancer is the most common gynecological malignancy in the Western world and EOC accounts for 90% of the cases [2]. Cisplatin is the first line chemotherapeutic agent for patients with ovarian cancers [3]. However, despite initial sensitivity to platinum-based chemotherapy, EOC often develop drug-resistance, which limits patient survival [3]. AKT survival signaling, which is a crucial regulator of cell proliferation, growth, survival and metabolism, had been implicated in the development of drug-resistance and the progression of EOC [5, 6]. There are emerging evidences for the involvement of AKT in controlling the apoptosis mechanism in response to cisplatin-induced DNA damage, promoting the cell survival and acquiring eventual cisplatin-resistance in EOC [5, 6]. DNA-PK was found to phosphorylate AKT at Ser473 directly in response to platinum, promoting resistance. The phosphorylated AKT exerts its biological functions via phosphorylation of its downstream targets, ultimately inhibiting apoptosis and promoting cell survival [5, 6]. Many studies had found that AKT activation is frequently observed in patients with relapse and therefore the potential role of AKT in the acquired cisplatin resistance may have significant clinical relevance [4–7]. Furthermore, Bcl-2 family proteins are key modulators of the intrinsic mitochondrial-mediated apoptotic pathway and regulation of the activity of some members within this family have been associated with AKT phosphorylation and chemosensitivity in EOC [2, 8, 9]. The mechanisms in which Bcl-2 family proteins interact to bring about the cisplatin-resistant phenotype of DNA-PK mediated AKT activation remains to be fully elucidated. This study therefore aims to examine the potential involvement of Bcl-2 proteins in AKT-regulated cell survival in response to cisplatin in cisplatin-sensitive and cisplatin-resistant EOC cells. Here we provide some preliminary data on the differential expression of Bcl-2 family protein according to AKT activity and give some insights into the possible mechanisms of AKT in developing cisplatin resistance.

To investigate the association of AKT inhibition and subsequent resensitisation to cisplatin with Bcl-2 family members, Western blotting was used to identify the potential downstream targets of TCN and NU7441 within this family in cisplatin-sensitive (PEO1) and its cisplatin-resistant counterpart (PEO4) cell lines. TCN is a selective potent AKT inhibitor and is found to inhibit AKT activation directly with high affinity. Although there is limited literature regarding the effect of NU7441 on AKT inhibition, the fact that DNA-PK can phosphorylate AKT at Ser473 in presence of cisplatin can be used to suggest that NU7441 can function as an indirect AKT inhibitor [19]). After Western blotting screening of the Bcl-2 family proteins and several other potential apoptotic proteins, we identified interesting targets and selected Bad and Bcl-2 for further assessments.

Firstly, multi-domain pro-apoptotic Bax and Bak are executors of apoptosis and their oligomerizations on the outer mitochondrial membrane are required for the release of cytochrome c and induction of apoptosis [18]. Previous studies have suggested that Bax rather than Bak promotes apoptosis in ovarian cancer cells and the data shown here also support this. Bak expression is not affected by AKT inhibition whereas an elevated level of Bax expression was detected in response to AKT inhibition in cisplatin-resistant PEO4 cells. This suggests the potential involvement of Bax in re-sensitizing PEO4 cells to cisplatin-induced apoptosis in response to AKT inhibition. However, this evidence is not sufficient to show that Bax is a direct downstream target for AKT activation and therefore Bax was not taken forward for further study in this project. Moreover, the limitation of Western Blotting is its inability to tell whether the protein is active in forming the pores on the mitochondrial membrane. Thus Bak expression may not be altered but it could still be functionally modulated upon treatments.

**Figure 6:**
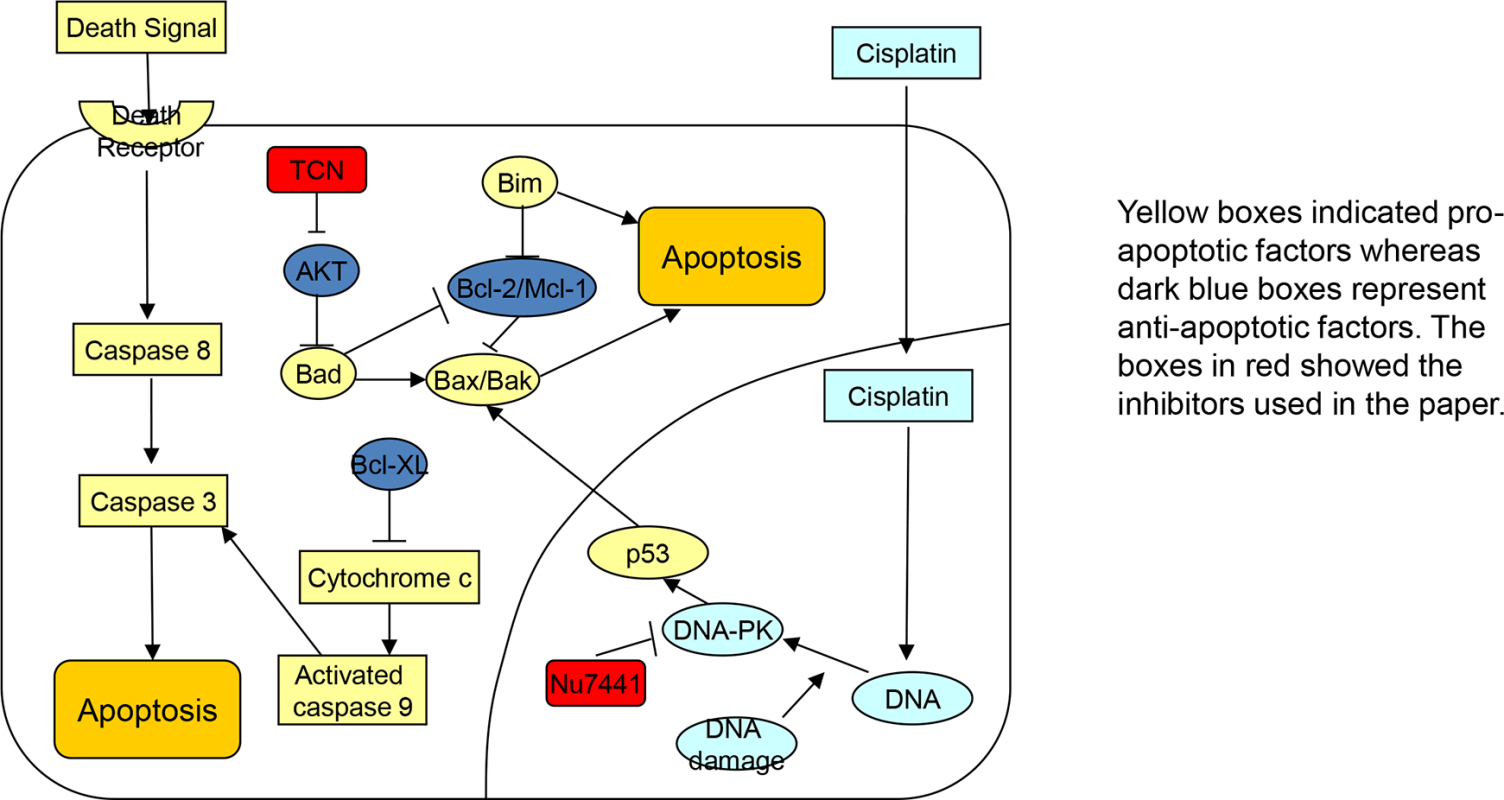
Schematic picture The signaling pathways related to combination treatment using cisplatin, DNA-PK inhibitor NU7441, and AKT inhibitor TCN.

Another pro-apoptotic subgroup of Bcl-2 family is the BH-3 only proteins, of which Bik, Bid, Bim, puma and Bad were assessed here using Western blotting. There are two models by which Bax may be activated by BH-3 only proteins, including direct activation and neutralization of the anti-apoptotic proteins [18]. Here BimEL expression was found to be up-regulated by the AKT inhibitor TCN in the presence of cisplatin in cisplatin-resistant PEO4 cells. This strongly supports a direct link between Bim expression and AKT phosphorylation in EOC cells. Moreover, expression of the more pro-apoptotic BimL was induced in PEO4 cells in response to AKT inhibition. Together, these results suggest that in addition to Bax, Bim is involved in the re-sensitization of chemoresistant EOC cells to cisplatin by AKT inhibitors. The data obtained from cisplatin-sensitive cells shown that the most pro-apoptotic isoform of Bim (Bims) was abundantly expressed especially in cells treated with cisplatin and TCN inhibitor. In the meantime, the BimEL level was significantly diminished by cisplatin. All these evidences indicate that Bim especially Bims plays a stimulatory role in apoptosis in response to cisplatin. (Figure 6).

The BH-3 only Bad protein, which is a known downstream substrate of the AKT phosphorylation, has also been implicated in determining the chemosensitivity in EOC [16]. The phosphorylation of Bad at Ser136 inactivates its pro-apoptotic activity and therefore we studied the expression variances of Bad protein and its phosphorylation at Ser136 in response to AKT inhibition and platinum treatment. Here in cisplatin-resistant PEO4 cells, Bad protein was found to be increased in response to AKT inhibition. Moreover, TCN-targeted AKT suppression prevented Bad phosphorylation at Ser136, which was seen in PEO4 cells treated with cisplatin. Together, these results suggest that inactivation of Bad may be an underlying cause of cisplatin resistance in PEO4 cells, and that AKT inhibition desensitizes these cells by both upregulation of Bad and reversal of its inactivation. Interestingly, Bad phosphorylation was found to be increased in PEO4 cells treated with NU7441, either alone or in combination with cisplatin. This suggests that inactivation of AKT activity via DNA-PK may activate an alternative re-sensitization mechanism, compared with direct inhibition of AKT. In cisplatin-sensitive PEO1 cells, Bad expression was not affected by AKT inhibition. However, cisplatin-induced Bad inactivation via phosphorylation was not prevented in cells co-treated with TCN or NU7441. This may partly explain why AKT inhibition does not increase the sensitivity of PEO1 cells to cisplatin. However, AKT inhibition alone decreased Bad phosphorylation in PEO1 cells. Together, this suggests that Bad does not have an important role in the apoptotic response of chemosensitive PEO1 cells to cisplatin. Given the potential importance of bad in regulating cisplatin-sensitivity in PEO4 cells, inhibition of Bad expression by siRNA knockdown was found to partially prevent cisplatin-induced apoptosis in transfected PEO4 cells. The incomplete repression of apoptosis implies that Bad alone is not sufficient for cisplatin-induced apoptosis in these cells, and that other protein are also involved in the induction of apoptosis. However, this suppression may also have arisen due to the cytotoxicity of the transfection reagents to the cells during the experiments. Although cells remained viable after 72 hr transfection as seen in the initial MTT assay, additional stresses of harvesting and reseeding may have accumulated and these stresses may cause widespread cell death at the 72 hr post-transfection time point. Thus the knockdown of Bad may not be involved in, or sufficient to prevent cell death in such instance. In addition, the use of a high amount of Bad siRNA (100 nM) may have contributed to cytotoxicity. However, this amount was chosen,although 50 nM Bad siRNA may have a lower cytotoxicity, the transfection efficiency was too low (50%) to demonstrate the influence of Bad siRNA knockdown on cell viability. This functionally highlights the potential pro-apoptotic role of Bad in cisplatin-resistant cells.

In comparison to Bim and Bad, expression of pro-apoptotic BH-3 only proteins Bik and Bid were not found to be regulated by AKT in the present study. Bik is rarely associated with AKT activation in literature but its expression has been shown to be necessary for caspase-8 activation. Bik expression was not affected by TCN- or NU7441-mediated AKT inhibition, however, its expression level was elevated in response to cisplatin treatment in both cisplatin-sensitive (PEO1) and cisplatin-resistant (PEO4) cells. Thus it is possible that Bik is involved in the apoptotic pathway directly activated by cisplatin in ovarian cancer cells but its activity is not induced by AKT signaling pathway. The lack of effect of cisplatin, TCN and NU7441 on Bid expression in PEO1 and PEO4 cells suggests that this protein is not involved in apoptotic pathways activated in these cells, and also that AKT does not regulate the activity of this protein. Unlike Bik and Bid, the expression of the pro-apoptotic BH-3 only protein may be regulated by AKT in PEO1 and PEO4 cells. However, the effect of AKT inhibition on Puma expression was found to be different in the cell lines, and was increased in PEO1 cells but decreased in PEO4 cells. Puma is a p53 inducible gene and acts as pro-apoptotic factor by binding to anti-apoptotic Bcl-2 [15]. A recent study has established that induction of Puma expression by cisplatin was abolished in p53-deficient SKOV3 cells whereas increased Puma expression would sensitize SKOV3 cells to cisplatin via down-regulation of anti-apoptotic Bcl-XL and Mcl-1 [15]. Moreover, the Puma-triggered Mcl-1 down-regulation can be associated with caspase-dependent cleavage. Therefore overexpression of Puma could potentially enhance the sensitivity to cisplatin in EOC by lowering the threshold set simultaneously by Bcl-XL and Mcl-1. Thus, as both PEO1 and PEO4 cells are p53 mutated the lack of effect of cisplatin on Puma expression may due to this. However, the mechanism by which AKT inhibition regulates Puma expression may be p53-independent, and remains to be investigated. From these aspects, the role of Puma in cisplatin-induced apoptosis and in determining the chemosensitivity remains complex and unclear.

Bcl-2 is an intriguing member within the Bcl-2 family that may be identified as a downstream target of AKT phosphorylation. In this study Western blot data suggested an association between AKT inhibition and suppression of anti-apoptotic Bcl-2 protein expression in both PEO1 and PEO4 cells. Bcl-2 expression was decreased by NU7441 regardless of the presence or absence of cisplatin. In addition, TCN also exerted a complete inhibitory effect on Bcl-2 expression in PEO4 cells when it was used in combination with cisplatin. Interestingly, phosphorylation of Bcl-2 at Ser70 (pBcl-2) was completely abrogated in PEO1 cells in response to cisplatin and AKT inhibition. And the phosphorylation of Bcl-2 at Ser70 was not detected in PEO4 cells, implying a potential regulatory role of Bcl-2 in cisplatin-sensitivity via its phosphorylation. Several studies speculated that pBcl-2 (Ser70) status is closely correlated with metastasis and the extent of malignancy in colorectal cancer and the absence of pBcl-2 (Ser70) is closely associated with poor survival in colorectal cancer [17]. Loss of pBcl-2 (Ser70) was more frequently recognized in the cases with advanced stage of lymph nodal metastasis and clinical stages in comparison to the poorly differentiated cases [17]. Considering this particularly with our evidences, it is convincible to assume that phosphorylation of pBcl-2 at Ser70 could play a similar inhibitory role in the inhibition of apoptosis in EOC. Therefore pBcl-2 (Ser70) can be suggested as biological marker for the extent of cisplatin-sensitivity in EOC and a novel prognostic indicator. It is also necessary to mention that pBcl-2 (Thr56) was not detected in this study and therefore we assume that no clear association of phosphorylation of Bcl-2 at Thr56 found according to present study.

In comparison, Bcl-XL expression was decreased in response to TCN in PEO1 cells only, and to NU7441 in PEO4 cells only. Together, these results indicate a greater role for Bcl-2 in the AKT-mediated regulation of cell survival pathways. Furthermore, the Western blot analysis on Mcl-1 expression can be interpreted alongside with the protein profiling results of Bcl-2. Intriguingly, inhibition of AKT increased Mcl-1 expression in PEO4 cells. This suggests that although AKT inhibition can inhibit expression of anti-apoptotic bcl-2, the cells may attempt to counteract this by increasing Mcl-1 expression as a possible cellular compensatory mechanism (Figure 6). Thus it is possible that cisplatin resistance in these cells requires suppression of more than one anti-apoptotic Bcl-2 family proteins. To further clarify the role of Bcl-2 and Mcl-1 in cisplatin-induced apoptosis, a BH-3 mimetic ABT-737, which has a high affinity for Bcl-2 and Bcl-XL but not Mcl-1, was used in both cisplatin-sensitive and cisplatin-resistant cell lines (Figure 6). Previous studies shown that ABT-737 triggered Bax/Bak-mediated apoptosis by targeting Bcl-2/Bcl-XL but ABT-737's inability to target Mcl-1 may confer resistance to cisplatin *in vitro* [22]. Here ABT-737 inhibitor was found to sensitize both PEO1 and PEO4 cells to cisplatin treatment, confirming that Bcl-2 has an important role in determining the cisplatin-sensitivity in EOC. Accordingly, ABT-737 in combination with cisplatin may be an effective strategy for enhancing the response of patients to cisplatin therapy.

In addition to the Bcl-2 family of proteins, the role of the apoptotic effectors caspase 8 and caspase 9 in the response of EOC cells to cisplatin and AKT inhibition was also assessed (Figure 6). Here the cleaved form of caspase 9 was only detected in PEO1 cells in response to cisplatin treatment. However, it is possible that the proteolytic activation of caspase 9, which occurs downstream of Bcl-2 proteins may only be detectable after treatment periods of greater than the 8 hrs time point which was used in this study. Similarly, no cleaved forms of caspase 8 were detected, but assessment of their expression after longer treatment times will be required to determine whether or not it is involved in the response of the cells to the drugs. However in support of a role for caspase 9 in cisplatin-induced apoptosis in PEO1 cells expression of X-linked inhibitor of apoptosis (XIAP), which prevents activation of caspase 9, was found to be decrease in cisplatin-sensitive PEO1 cells treated with cisplatin. Apparently, more assessment is needed to give more information on XIAP molecular actions during apoptosis. Together with the data regarding Bcl-2 protein expression, these results suggest the intrinsic apoptotic pathway is the main mechanism by which apoptosis is induced in cisplatin-sensitive PEO1 cells treated with cisplatin (Figure 6).

Taken together, our results suggest that Bcl-2 family proteins are regulators of drug resistance. This study provides rational to support using a combination of cisplatin and ABT-737 to treat cisplatin-resistant ovarian cancer.

## MATERIALS AND METHODS

### Materials and chemicals

AKT inhibitor TCN, DNA-PK inhibitor NU7441, and Bcl-2 inhibitor ABT-737, were obtained from Berry and Associates (Devon, UK), KuDOS Pharmaceuticals (Cambridge, UK) and Allan Richardson (London, UK), respectively. They were dissolved in DMSO. Cisplatin (1 mg/ml in PBS) was obtained from the Pharmacy Department, Hammersmith Hospital, London, UK. All other chemicals were purchased from Sigma-Aldrich (Dorest UK), and all solutions were prepared and diluted using distilled water.

### Cell culture

PEO1, PEA1 and PEO14 are platinum-sensitive cell lines while their intra-patient paired variants, PEO4, PEA2 and PEO23, are platinum-resistant. They were all generated from ascites fluid taken from patients with ovarian cancer before cisplatin treatment (PEO1, PEA1, PEO14) and after development of chemoresistance (PEO4, PEA2, PEO23) [23]. These cell lines were maintained in RPMI 1640 medium (Sigma-Aldrich, St. Louis, MO, USA) supplemented with 10% fetal calf serum (FCS) (First Link, UK), 50 U/ml penicillin/streptomycin (Invitrogen, Paisley, UK), 2 mM L-Glutamine in humidified incubator at 37°C with 5% CO_2._

SKOBS v1.2, SKOBS 3.5, BKS 2.1 and P95R-3.4 cell lines are SKOV-3 derived stable-transfected cell lines, expressing 0×OPCML (empty vector), 3×OPCML, 30 × OPCML and 30× P95R-OPCML, respectively. These cells were grown as before but with medium also supplemented with Zeocin (125 μg/ml, Invitrogen, (California, USA)).

sh339-24 (OPCML short-hairpin RNA knockdown cell line) and PLKO-1.3 are OSE-C2 derived cell lines, representing 95% knockdown of OPCML and 1×OPCML, respectively. These two cell lines were maintained in RPMI 1640 medium supplemented with 10% FCS, 2 mM L-Glutamine and 3 μg/ml Puromycin (Invitrogen, (California, USA) at 33°C with 5% CO_2._

### Drug treatments

When required for drug treatment, cells were harvested by trypsinisation and cell counting was carried out using a hemacytometer. The trypsinized cells were centrifuged at 15,000 rpm for 5 mins and resuspended by pipetting up and down thoroughly. Then the cells were counted using a hemacytometer (Thermo Scientific, USA) following manufacturer's instructions. Cells were seeded at the required density and allowed to adhere overnight. When required for detected of phospho-proteins, cells were serum starved overnight by incubating in serum-free medium. Culture medium was then removed, and replaced with the required concentrations of drugs, or vehicle control. Treated cells were incubated under the appropriate conditions and the required time period.

### SDS-PAGE and Western blotting

Following drug treatment, cells were washed once with ice-cold phosphate-buffered saline (PBS) and lysed using 30 μl Radioimmunoprecipitation (RIPA) buffer [50 mM 1×TBS, 0.1% Nonidet P-40, 0.5% Sodium Deoxycholate, 0.1% SDS, 0.004% Sodium Azide, PMSF, Sodium orthovanadate and 10 μl/ml 100× phosphatase inhibitor cocktail] (Santa Cruz Biotechnology, CA, USA), as per manufacturer's instructions, or 200 μl RPPA lysis buffer [50 mM Hepes (pH 7.4), 150 nM NaCl (Thermo Scientific), 1 mM EGTA, 10 mM Sodium Pyrophosphate, 100 μM NaF, 10% Glycerol, 1.5 mM MgCl_2_, 1% Triton X-100 and 10 μl/ml 100× phosphatase inhibitor cocktail (Santa Cruz Biotechnology, Texas, USA)]. Then the cells were left on ice for 15 mins and harvested with a cell scraper. The lysate was then centrifuged at 13,300 rpm for 10 mins at 4°C and the supernatant was collected. The protein concentration of each sample was examined by Bicinchoninic Acid (BCA) protein assay (Thermo Scientific, CA) following the manufacturer's instructions.

25 μg protein was added to an equal volume of 2× loading buffer [0.5M Tris-HCl (pH6.8), 20% Glycerol, 10% SDS, 2% 2-mercaptoethanol and 0.1% Bromophenol Blue] and boiled for 5 minutes at 90°C. The denatured proteins were resolved by 8% or 12% sodium dodecyl sulphate polyacrylamide gel electrophoresis (SDS-PAGE) using a Mini-Protean electrophoresis system (Bio-Rad, Berkeley, CA, USA). The separated proteins were then transferred onto nitrocellulose membrane (Bio-Rad, Berkeley, CA, USA) at 100 volts for 1 hr using a Mini-TransBlot system (Bio-Rad, Berkeley, CA, USA). The membranes were then blocked with 5% skimmed milk in tris-buffered saline (TBS) containing 0.1% Trition-X (TBS-T)or 5% BSA in TBS-T for detection of phospho-proteins, for 1 hour at room temperature with shaking. The membranes were incubated with primary antibodies (Table 1) at 4°C overnight with shaking. The blots were then washed three times for 5 mins each, with TBS-T prior to incubation with appropriate horseradish peroxidase (HRP)-conjugated secondary antibody (Table 1) for 1 hr at room temperature with shaking. The blots were washed as before and proteins were visualized using Millipore^™^ Immobilon^™^ Western Chemiluminescent HRP Substrate system (Millipore Corporation, Billerica, MA, USA) as per manufacturer's instructions. The blots were developed on photography film using Kodax SRX SRX2200 (Rochester, NY, USA).

### siRNA transfection

The transfection with Bad small interfering RNA (siRNA) (5′→3′ sense: GGAGGAUGAGUGACG AGUUtt; 5′→3′ anti-sense: AACUCGUCACUCAUCCUC Cgg) (Applied Biosystems/Ambion, USA) was performed using antibiotic-free RPMI media containing 10% FCS and 2 mM L-Glutamine. PEO4 cells were seeded at 1 × 10^6^ cells/well in 6-well plate and left in incubator overnight. Cells were transfected by the addition of Optimem medium (400 μl, Invitrogen) containing 100 nM Bad siRNA or siGenome Lamin A/C control siRNA (Dharmacon, Denver, Colorado, US) in the presence of 0.1% Transfection Reagent-1 (Dhramacon). After 48 hrs, a second transfection was carried out and the cells were incubated for a further 24 hrs. Transfected cells were then washed with PBS and trypsinized using 1 × Trypsine as previously described (Section 4.3). Cells were then counted and seeded in 6-well plate and 96-well plates for Western Blotting analysis, MTT and Caspase 3/7 Assays, respectively.

### MTT assay

Cells were seeded at a density of 20,000 cells /well in a 96-well plate and incubated overnight to allow cell attachment. The transfected cells were treated with 25 μM cisplatin or vehicle control for 24 hours. 10 μl of MTT solution (3 mg/ml MTT in PBS) was added to each well. After 2-hr incubation in the incubator, equal volume (i.e. 60 μl) of MTT STOP solution (10% SDS in 0.01% HCl) was added to the mixture and the plate was wrapped with foil, and incubated at room temperature overnight with shaking. Absorbance was measured on a microplate reader (Spectra Max 190) at 570 nm.

### Caspase 3/7 assay

The cells were seeded at 20,000 cells/well in a white opaque 96-well plate (PerkinElmer, Singapore) and the plate was left in incubator overnight. Cells were treated with 25 μM cisplatin or vehicle for 24 hrs. Caspase-Gloâ 3/7 Substrate (Promega, USA) was added as per manufacturer's instructions and the plate was incubated at room temperature for 1 hr. Luminescence was measured using a LUMIstar model luminometer (BMG LabTech) using OPTIMA software (BMG LabTech).

### Data analysis

Statistical analysis was carried out using Microsoft Excel 2001 and data are presented as mean ± standard error of the mean (SEM) combining three experimental repeats. The Western blot results were analyzed using densitometry (Image J software) where appropriate and the values of the protein densities were normalized to their corresponding beta-tubulin loading control to compare the variances of protein expression under specified conditions.
