# Correction: The involvement of Bcl-2 family proteins in AKT-regulated cell survival in cisplatin resistant epithelial ovarian cancer

**DOI:** 10.18632/oncotarget.27376

**Published:** 2020-01-28

**Authors:** Yan Dai, Shiguang Jin, Xueping Li, Daxin Wang

**Affiliations:** ^1^ The Second Xiangya Hospital of Central South University, Changsha, China; ^2^ Clinical Medical College, Yangzhou University, Yangzhou, China; ^3^ Medical Research Centre, Northern Jiangsu People’s Hospital, Yangzhou, China; ^4^ Nanjing Hospital Affiliated to Nanjing Medical University, The First Hospital of Nanjing, Nanjing, China

**This article has been corrected:** Due to errors during image assembly, the western blotting results of b-tubulin for Bax and Bak in PEO1 cells, shown in Figure 2A, were accidental duplicate images. The corrected Figure 2A is shown below. The authors declare that these corrections do not change the results or conclusions of this paper.

Original article: Oncotarget. 2017; 8:1354–1368. 1354-1368. https://doi.org/10.18632/oncotarget.13817

**Figure 2:**
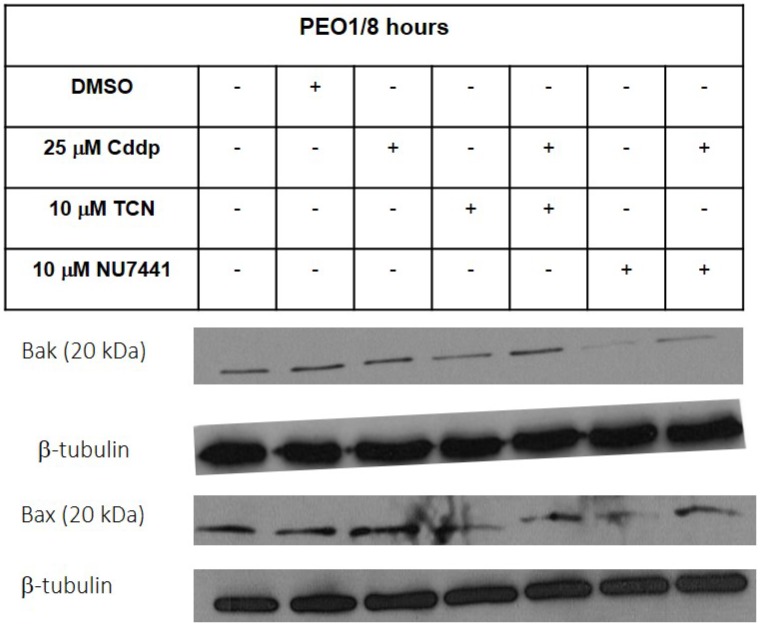
Expression of pro-apoptotic Bcl-2 family proteins in PEO1 and PEO4 cells in response to treatment with cisplatin, TCN and NU7441.

Cisplatin-sensitive PEO1 and cisplatin—resistant PEO4 cells were treated as indicated. Cell lysates were prepared and were separated by 12% SDS-PAGE gel as described in Materials and Methods. Western Blotting was carried out to examine expression of (**A**) multi-domain pro-apoptotic Bax and Bak.

